# Alcohol policy enforcement and changes in student drinking rates in a statewide public college system: a follow-up study

**DOI:** 10.1186/1747-597X-5-18

**Published:** 2010-08-04

**Authors:** Sion K Harris, Lon Sherritt, Shari Van Hook, Henry Wechsler, John R Knight

**Affiliations:** 1 Center for Adolescent Substance Abuse Research, Children's Hospital Boston, 300 Longwood Ave., Boston, MA 02115, USA; 2 Department of Society, Human Development and Health, Harvard School of Public Health, 677 Huntington Ave., Boston MA 02115, USA

## Abstract

**Background:**

Heavy alcohol use among U.S. college students is a major contributor to young adult morbidity and mortality. The aim of this study was to examine whether college alcohol policy enforcement levels predict changes in student drinking and related behaviors in a state system of public colleges and universities, following a system-wide change to a stricter policy.

**Methods:**

Students and administrators at 11 Massachusetts public colleges/universities completed surveys in 1999 (N of students = 1252), one year after the policy change, and again in 2001 (N = 1074). We calculated policy enforcement scores for each school based on the reports of deans of students, campus security chiefs, and students, and examined the correlations between perceived enforcement levels and the change in student drinking rates over the subsequent two year period, after weighting the 2001 data to adjust for demographic changes in the student body.

**Results:**

Overall rates of any past-30-days drinking, heavy episodic drinking, and usual heavy drinking among past-30-days drinkers were all lower in 2001 compared to 1999. School-level analyses (N = 11) found deans' baseline reports of stricter enforcement were strongly correlated with subsequent declines in heavy episodic drinking (Pearson's r = -0.73, p = 0.011). Moreover, consistently high enforcement levels across time, as reported by deans, were associated with greater declines in heavy episodic drinking. Such relationships were not found for students' and security chiefs' reports of enforcement. Marijuana use did not rise during this period of decline in heavy drinking.

**Conclusions:**

Study findings suggest that stronger enforcement of a stricter alcohol policy may be associated with reductions in student heavy drinking rates over time. An aggressive enforcement stance by deans may be an important element of an effective college alcohol policy.

## Background

Numerous national surveys have shown that heavy alcohol use among U.S. college students is a pervasive and enduring public health problem. According to the Monitoring the Future Survey and the Harvard School of Public Health College Alcohol Study (CAS), about two in five students attending 4-year colleges in the U.S. report engaging in heavy episodic drinking, defined as >=5 drinks for males and >=4 drinks for females consumed on a single occasion during the prior two weeks [1,2]. The heavy drinking rate among college students is higher than that of their non-college-attending peers, and has shown greater resistance to change throughout the surveyed years [1,3,4].

Heavy alcohol use is a major contributor to morbidity and mortality among U.S. college students. Hingson et al. [3] estimated that, in 2001, nearly 600,000 college students were injured because of drinking and more than 1500 college students died from alcohol-related motor vehicle crashes and other alcohol-related injuries. Two such student deaths occurred in Massachusetts in the fall of 1997 and were highly publicized [5]. One student died from alcohol poisoning after participating in a fraternity event and the other died from an alcohol-related injury. These deaths prompted the Massachusetts Board of Higher Education (MBHE), the agency governing the statewide system of public colleges and universities, to adopt a new, more restrictive alcohol policy for all schools under its authority. The new policy included eight components: (1) restricting alcohol to specific, supervised locations; (2) requiring advance registration of all social events involving alcohol; (3) restricting "legal" possession of alcohol to separate residence halls for students age 21 or older; (4) providing alcohol education and prevention programs; (5) establishing procedures for enforcement of all federal, state, local, and campus regulations; (6) requiring that colleges work with neighboring cities and towns to enforce alcohol laws; (7) new sanctions on student violators, up to and including expulsion from the college; and (8) parental notification of all alcohol policy violations by underage students. This statewide action provided a unique opportunity to study an unfolding natural experiment involving multiple schools and thousands of students. We conducted an initial assessment in 1999 to examine heavy drinking behavior among students attending schools under the purview of the MBHE, and school administrator and student reactions to the new policy one year after its adoption. Since the effectiveness of any policy depends on the strength of its implementation and enforcement, we particularly focused on the level of policy enforcement across the schools, as perceived by the dean of students, campus security chief, and students at each school, and assessed the relationship between policy enforcement and heavy drinking rates.

Results of that previous study [6] indicated that heavy episodic drinking rates and perceived level of policy enforcement varied widely across the 11 schools, and after adjustment for student demographic differences across schools, the heavy drinking rates for students living *on campus *were significantly and negatively correlated with the level of policy enforcement as reported by the chief of campus security at each of the schools. Interestingly, heavy drinking rates had low to moderate positive correlations (not statistically significant) with enforcement levels reported by the dean of students at each school and by the students. We hypothesized that these positive correlations may be a reflection of deans having to discipline more students at schools with higher drinking rates, and of students who drink being more likely to encounter alcohol policy enforcement. Data for this study, however, were cross-sectional and we could not determine the temporal relationship between alcohol policy enforcement and student drinking rates.

The current study extends our previous study by examining longitudinally how student drinking and related behaviors changed over the subsequent two-year period (1999-2001), and whether reported levels of alcohol policy enforcement related to the observed changes. Specifically, we first examined how policy enforcement levels in the initial year predicted *subsequent *change in commonly assessed college drinking rates, i.e., any past-12-months drinking, any past-30-days drinking, recent heavy episodic drinking, usual heavy episodic drinking among drinkers, and uptake of heavy episodic drinking in college. We hypothesized that higher baseline enforcement levels would predict decreases in student drinking rates over time. We then examined how reported policy enforcement levels *across *the two years related to student behavior changes during the same time period. We hypothesized that schools that *started *with higher levels of alcohol policy enforcement in the initial year, and that reported *consistently *higher levels of enforcement across time, would have, on average, a greater decline in student drinking rates compared to schools that reported lower enforcement levels over time.

With this longitudinal study, we also had the opportunity to look at possible unintended consequences of a more restrictive alcohol policy. We examined the relationship between alcohol policy enforcement levels and change over time in student marijuana use rates, as there might be a transfer from alcohol to other types of substance use due to greater restrictions on alcohol. In addition, we examined how alcohol policy enforcement levels related to changes in rates of student drinking and driving or riding with a driver who had been drinking, since a more restrictive campus environment may result in more students choosing off-campus venues for drinking.

## Methods

This longitudinal study consisted of two assessments conducted two years apart (1998/1999 and 2000/2001) of the behavior and perceptions of students and administrators at 11 public colleges and universities in Massachusetts. The schools, baseline sample, and data collection instruments and method are described in detail in a previous report [6].

### Student survey

We slightly shortened the student survey used in 1999 (Time 1 [T1]) to assess student behaviors and alcohol policy enforcement perceptions, and re-administered it in 2001 (Time 2 [T2]) at the same time of year as the 1999 survey. For both cohorts, we sent out the first mailing during the week of April 5^th^, and a second mailing during the first week of May. We chose this timing in order to avoid having spring vacation included in the prior two-week timeframe for survey responses, and to reach students before the end of the school year. Our surveys were also timed to coincide with the administration of the national CAS survey.

As in the 1999 survey, the registrar at each school randomly selected 225 full-time undergraduates from the total enrollment who were then mailed a survey. The random sampling method was the same as that used in all of the national CAS surveys [7]. In brief, registrars were instructed to draw every *x*th student name (number determined by enrollment size of school), beginning at a random starting point, from the enrollment roll. To protect student confidentiality, the survey included no identifying information except for a numeric code identifying the school. We therefore could not link data for individual students across the two surveys. In the 2001 survey, 2,222 surveys were deliverable and 1,074 were returned completed, for a 48.3% overall response rate (range for individual schools, 38.4% to 54.3%). This was a lower overall response rate than in 1999 (56%), mirroring a declining trend in response rates seen in the CAS across the same time period (59% to 52%) [2]. To explore the degree to which differential non-response might explain differences in drinking rates across schools and across years, we examined correlations between school response rates and heavy drinking rates at each time point. We found low correlations in both baseline (Pearson's r = 0.175, p = .606) and follow-up years (Pearson's r = 0.273, p = .418).

### Administrator surveys

To assess school administrators' perceptions of alcohol policy enforcement, we mailed identical questionnaires in the fall of 1998 (Time 1), and again in the fall of 2000 (Time 2), to the dean of students and chief of campus security at each school. The questionnaire assessed administrators' views on the new alcohol policy, and their perception of policy implementation and enforcement at their school. These surveys included no identifying information except for a numeric school code allowing linkage of administrator and student surveys. In both survey administrations, all 11 deans, and 10 of 11 campus security chiefs, returned questionnaires. The school with missing security chief data was not the same across the two time points, resulting in only 9 schools having longitudinal data from security chiefs. Because of personnel changes across the two years, 6 of 11 schools had the same dean responding at both time points, and 7 of 9 had the same campus security chief. Four schools had both the same dean and campus security chief respondents across the two time points.

## Measures

### Perception of Enforcement Level

Security chiefs and deans responded to 14 identical survey items. Nine items assessed their perception of alcohol policy enforcement level (1 = "rarely enforced," 2 = "enforced when violations are blatant or reported," or 3 = "aggressively enforced in all circumstances") at student gatherings in dorm rooms, dorm parties, fraternity/sorority parties, on-campus dances and concerts, intercollegiate and intramural sports events, pre- and post-game parties, and homecoming celebrations. Six items assessed the regularity of use (1 = "not used," 2 = "occasionally used," or 3 = "regularly used") of specific procedures to limit on-campus drinking (e.g., stopping and searching students entering residences, checking student IDs). We summed response scores for these 14 items and computed a percent-of-total score by dividing the sum by the highest possible total score for that school (some schools did not have fraternities/sororities). We calculated separate scores for deans and security chiefs; higher scores indicated greater perceived enforcement.

We assessed students' perceptions of enforcement using 11 items. Students reported how likely (1 = "Very Unlikely" to 5 = "Very Likely) they thought an underage student would get caught if they drank alcohol in a dorm room, at a dorm party, at a fraternity/sorority party, or at an intercollegiate sports home event; and how likely a student caught violating the alcohol policy would experience specific consequences (e.g., get "written" up, fined, have on-campus housing revoked, or face other disciplinary action). Students also gave a global rating of the strength of enforcement at their school ("Not enforced at all," "Weakly enforced," "Enforced," "Strongly enforced," and "Don't know school's policy"). Since a sizable proportion of respondents gave the "don't know" response (16%), we collapsed the "not enforced" and "don't know school's policy" into a single category. Students' lack of awareness of the school's policy may be an indication that they had few or no encounters with enforcement actions. These 11 items had strong internal consistency reliability (Cronbach's alpha = 0.79) and we calculated a sum score for students who answered at least 80% of items (9 items). If data were missing for an item (up to 2 items), mean imputation was used to replace missing data. For school-level analyses, we computed the mean of the students' sum scores.

We found little or no agreement among the three different perspectives on alcohol policy enforcement; inter-"rater" correlation coefficients at T1 were -0.26 between deans and campus security chiefs, -0.04 between deans' and students' scores, and 0.25 between campus security chiefs and students. On the other hand, there was stronger consistency in reports from the same respondent across time (T1 to T2). When the same dean provided data at both time points (n = 6), deans' enforcement scores were highly consistent across the two years (intra-class correlation coefficient [ICC] = .79). Security chiefs' enforcement reports, however, showed more variability over time, with a more moderate correlation (ICC = .37) across data given by the same chief (n = 7). Not surprisingly, there was little consistency across the two years in deans' or security chiefs' enforcement scores when reported by different individuals. There was no difference between years in mean student enforcement scores.

### Student Behavior Trends

We assessed student drinking using standard "drink" definitions (e.g., 12 oz can or bottle of beer) and timeframes from the CAS: (a) any *past-12-months drinking*, (b) any *past-30-days drinking*, (c) any *past-2-weeks heavy episodic drinking *(HED) defined as >=5 drinks per occasion for males and >=4 for females [8], and (d) *usual heavy episodic drinking *(UHED) among those reporting drinking in the past 30 days, i.e., *usually *drinking >=5 drinks for males and >=4 drinks for females whenever they drink. In addition, we asked students whether they had ever engaged in heavy episodic drinking during their last year of high school and examined the change over time in the proportion of students who reported HED in college but *who had not engaged in HED in their last year of high school *("never" for high school item and >=one occasion of past-2-weeks HED).

Finally, we also assessed any past-30-days use of marijuana and any past-30-days driving while intoxicated or riding with an intoxicated driver (DWI/RWID). All survey items came from the national CAS survey [9].

### Data analysis

We scanned student questionnaires directly into an electronic data file, and we manually entered administrator questionnaire data twice into MS Excel, compared the two files, and corrected any discrepancies. We used SUDAAN^® ^9.0 software for analyses involving individual student data to correctly account for the cluster (school) sampling design in our precision estimates, and SPSS^® ^15.0 software for analyses involving school as the unit of analysis.

We standardized the two years' samples by applying weights derived from the T1 sample demographic profile for each school (gender, age group [<21 yrs, 21+ yrs], race group [White, non-White], on/off-campus housing status) to the T2 follow-up data for that school. We then conducted chi-square tests (all with one degree of freedom) to assess whether the weighted rates of interest (past-12-months drinking, past-30-days drinking, past-2-weeks heavy episodic drinking, heavy episodic drinking as a usual pattern among past-30-days drinkers, uptake of heavy episodic drinking in college, past-30-days driving/riding after drinking, and past-30-days marijuana use) were significantly different between the two years. We dichotomized race into White and non-White due to small percentages in the individual non-White categories (no category had more than 5%). We also conducted stratified chi-square analyses to assess trends in drinking rates by gender, age group, and housing status.

We then calculated a change score for each student behavior variable for each of the 11 schools by taking the absolute rate difference between the two years, dividing by the T1 rate, and multiplying by 100 to generate a percentage change score. The score was positive if the rate had gone up over time, and negative if the rate had gone down (e.g., T1 rate = 50%, T2 rate = 40%, change score = -20%).

To control for multiple comparisons in the subgroup analyses, we used the Benjamini-Hochberg (B-H) procedure, a sequential approach to controlling the false discovery rate (the expected proportion of erroneous rejections among all rejections of the null hypothesis) that is gaining preference over the more conservative Bonferroni correction [10-12]. In the B-H procedure, the observed p-values are ordered in descending size and compared to an ordered list of critical values generated from a linear interpolation from α/2 to (α/2)/*m *where *m *is the number of comparisons.

To analyze the relationship between *initial *alcohol policy enforcement levels and *subsequent *change in our student behavior rates of interest, we calculated correlation coefficients (Pearson's r) between T1 enforcement scores as reported by deans, security chiefs, and students, and the drinking rate change scores (N = 11). To assess how administrator-reported policy enforcement levels *over time *related to student drinking rate changes, we first identified the major *trajectories *of enforcement using hierarchical cluster analysis. Cluster analysis has previously been used to identify longitudinal trajectory profiles [13] and is a classification method which allows for the empirical identification of groups of cases that share similar profiles across characteristics of interest [14]. For each type of administrator, we entered their enforcement scores for both years into a hierarchical cluster analysis in SPSS^® ^v. 15.0, using squared Euclidean distance as the measure of similarity/distance across parents and Ward's method for combining clusters [15]. We chose the number of clusters by examining the agglomeration schedule and identifying the "elbow" in the curve of the distance measure across the cluster-joining steps; i.e., we looked for the point where the distance coefficient made a sudden jump in size. Based on this method, we decided to evaluate 2-3 clusters and examined for each cluster solution how distinct the groups were with respect to enforcement trajectories. We determined that 2 clusters for deans, and 3 clusters for security chiefs, were the optimal solutions due to the distinctiveness and interpretability of the profiles and the adequacy of cell sizes for subsequent analyses. We then used one-way analysis of variance (ANOVA) to compare the mean percent change over time in student drinking rates across the cluster groups.

## Results

### Sample Demographics

In 1999 and 2001, the student sample was majority female and white non-Hispanic (see Table 1), approximately half of respondents were under the legal drinking age of 21 years, about half lived on-campus, and <5% reported being members of a fraternity or sorority. The 2001 student sample (N = 1,074) had fewer Hispanic and more "Other" race students, and a higher proportion reporting heavy drinking during their last year of high school. Therefore, we standardized the follow-up sample using weights derived from the baseline sample to correct for the effect that demographic differences may have had on student drinking trends over time, and we report adjusted rates in Table 2.

**Table 1:** Demographic characteristics of the Massachusetts public college and university student samples, 1999 and 2001.

	*1999 sample* *(N = 1,252)*		*2001 sample* *(N = 1,074)*		*Chi-square* *statistic (df)*
*Characteristic*	*n*	*%*	*n*	*%*	***p-value***^**c**^
Gender					
Male	511	40.9	417	39.0	0.85 (1)
Female	737	59.1	651	61.0	.510
Race/Ethnicity					
White non-Hispanic	1071	88.4	929	87.4	6.1 (3)
Black non-Hispanic	34	2.8	28	2.6	.013
Hispanic	62	5.1	34	3.2	
Other race/ethnicity	45	3.7	72	6.8	
Age					
<21 years	638	51.0	502	47.0	0.86 (1)
>=21 years	612	49.0	567	53.0	0.38
Year in School					
Freshman	285	22.9	259	24.3	2.94 (5)
Sophomore	310	24.9	225	21.1	.069
Junior	312	25.0	254	23.8	
Senior	257	20.6	253	23.7	
>=5th year undergraduate	83	6.7	76	7.1	
Type of Residence					
On-campus	616	49.7	574	53.9	1.37 (1)
Off-campus^a^	624	50.3	490	46.1	.269
Fraternity/Sorority Member	50	4.0	30	2.6	4.68 (1) .056
Heavy Drinking in High School^b^					
No	729	58.8	581	54.7	5.31 (1)
Yes	510	41.2	481	45.3	.044

a Includes off-campus house or apartment, fraternity or sorority house, and other.

b Ever having >=5 drinks in a row (or >=4 for females) during last year of high school.

c Chi-square p-value estimates generated using SUDAAN^® ^9.0 software to account for sampling design effects.

**Table 2:** Comparisons^a ^of 1999 and 2001 rates of student drinking and related behaviors at eleven Massachusetts public colleges/universities.

	1999 (N = 1252) % (95%CI)	**2001**^**b**^ (N = 1074) % (95%CI)	**Chi-square**, p-value
Any drinking in past 12 months	87.9 (84.7-91.1)	86.3 (81.3-91.3)	0.46, .515
Any drinking in past 30 days	75.5 (71.6-79.4)	70.4 (64.8-76.0)	8.35, .016
Heavy episodic drinking^c ^in past 2 weeks	54.9 (49.3-60.5)	50.6 (45.2-56.0)	5.67, .039
Usual heavy episodic drinking *(among past-30-days drinkers)*^d^	67.1 (63.0-71.3)	56.8 (54.1-59.5)	16.27, .002
Heavy episodic drinking in past 2 weeks *among those reporting no heavy drinking in high school *(Uptake)	37.7 (32.7-42.7)	30.9 (24.0-37.8)	2.72, .130
Drove after drinking/rode with drunk driver in past 30 days	47.6 (43.8-51.4)	49.6 (44.5-54.7)	0.64, .442
Any marijuana use in past 30 days	22.6 (18.2-27.0)	22.2 (17.8-26.6)	0.04, .849

^a ^Chi-square tests were conducted, all with one degree of freedom, comparing 1999 and 2001 rates.

^b ^Rates reflect direct standardization to the 1999 sample profile with respect to gender, age, White/non-White race, and on/off-campus housing status.

^c ^Defined as >=5 drinks in one occasion for males, >=4 drinks for females.

^d ^Analysis subsample n = 837 for 1999, n = 760 for 2000 (unweighted). "Usual" heavy episodic drinking defined as *usually *engaging in heavy episodic drinking whenever they drink.

### Trends in Student Behaviors

There was no change in the overall rate of any *past-12-months drinking *for the total sample or for any demographic subgroup we examined (underage/legal-age, on-campus/off-campus residents, males and females), with the vast majority of respondents (86.3%) reporting past-12-months drinking in 2001 (Table 2).

On the other hand, the rates for any *past-30-days drinking *and *usual heavy episodic drinking *(UHED) among past-30-days drinkers declined significantly between the two survey years (Table 2). Past-30-days (i.e., current) drinking rates declined overall and specifically among underage students (72.5% vs. 63.0%, chi-square = 12.97, df = 1, p = .005) and on-campus residents (81.4% vs. 74.4%, chi-square = 8.54, df = 1, p = .015), but not among legal-age (78.6% vs. 80.1%) or off-campus residents (70.1% vs. 70.4%).

UHED rates among past-30-days drinkers showed dramatic and significant declines overall (Table 2), and across all age (underage/legal-age) and housing (on-campus/off-campus) subgroups. There was a gender interaction, however, with rates showing a significant decline among female students (69.2% to 54.1%, chi-square = 11.85, df = 1, p = .006), but not among male students (63.7% to 59.9%).

There was a declining trend in *past-2-weeks HED *(p = .039 was marginal after correction for multiple comparisons). In subgroup analyses, declining trends were seen among underage students (56.0% vs. 45.8%, chi-square = 7.88, df = 1, p = .019) and on-campus residents (65.2% vs. 56.1%, chi-square = 8.16, df = 1, p = .017) after multiple comparisons correction, but not among legal-age students (53.7% vs. 48.9%) or those living off-campus (45.1% vs. 43.7%).

*Uptake *of HED behavior in college showed a declining trend over time (Table 2), but the difference was not statistically significant. Uptake rates decreased marginally among underage students (36.7% vs. 24.7%, chi-square = 4.61, df = 1, p = .057) and on-campus residents (49.3% vs. 37.1%, chi-square = 5.36, df = 1, p = .043). Uptake rates showed little change among legal age students (38.7% vs. 38.5%) or among off-campus residents (27.3% vs. 31.9%).

We found no overall difference between years in the percentage of students reporting any past-30-days DWI/RWID. However, there was an increasing trend between survey years in the percent of *on-campus residents *reporting past-30-days DWI/RWID (44.1 vs. 49.9, chi-square = 4.14, df = 1, p = 0.069). There was no such trend among off-campus residents. We found little change in past-30-days use of marijuana for the total sample, or for any subgroup examined.

### Alcohol Policy Enforcement and Student Drinking Trends

#### Initial policy enforcement levels and drinking rate changes

**Figure 1:**
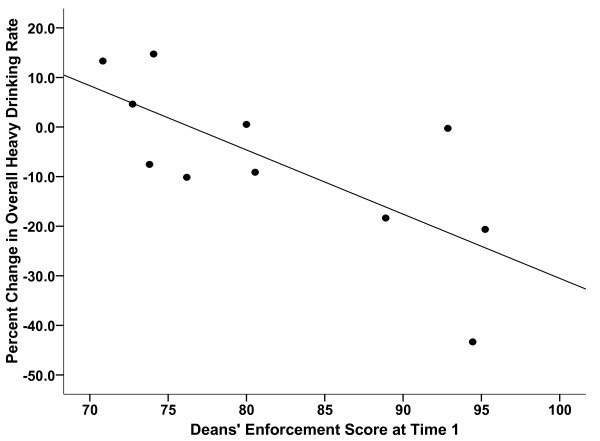
**Scatterplot of deans' enforcement score at baseline by percentage change in the overall student heavy episodic drinking rate between initial assessment and two-year follow-up**.

We found moderate to strong *negative *correlation coefficients for the association between T1 *deans' *enforcement scores and change in student drinking rates over the following two years. (Table 3) In particular, the correlation was strong between baseline deans' scores and changes in student HED rates. The relationship appears linear, with higher T1 enforcement scores associated with greater declines in the HED rate. (Figure 1) Removing the one outlier school (-43.3% change) reduced the correlation coefficient only slightly (r = -0.65, p = 0.04). In contrast, baseline security chiefs' enforcement scores tended to have small to moderate-sized *positive *correlations with subsequent drinking rate changes (Table 3); i.e., higher baseline scores were associated with less decrease in student drinking prevalence over time. The correlations between baseline *students' *reports of enforcement and drinking change scores were generally weak.

**Table 3:** Correlations between reported alcohol policy enforcement levels measured at baseline and changes between baseline and two-year follow-up in student drinking rates. (Pearson's r coefficients).

	Policy Enforcement Report		
	**Deans** **(n = 11)**	**Campus** **Security Chiefs** **(n = 10)**	**Students** **(n = 11**^**a**^**)**

Change in past-30-days drinking rate	-.30	.22	.30
Change in past-2-weeks heavy episodic drinking rate	-.73*	.31	.10
Change in usual heavy episodic drinking rate *among past-30-days drinkers*	-.36	.21	-.05
Change in past-2-weeks heavy episodic drinking rate *among those reporting no heavy drinking in high school*	-.45	.38	.04

Two-tailed significance p < 0.05

^a ^The mean student enforcement score for each school was correlated with its percent change in student drinking rate.

#### Policy enforcement levels over time and drinking rate changes

Cluster analysis of deans' enforcement scores over time identified two groups (see Table 4), a deans' score-based "higher enforcement" (D-HE) group of 6 schools and a "lower enforcement" (D-LE) group of 5 schools. The D-HE group was characterized by mean enforcement scores >=80 across both years, while the D-LE group had mean scores <80 over both years. Comparison of these two groups showed a significant difference in the mean percent change in past-2-weeks HED rate, with a mean -18.5% change for the D-HE schools compared to a mean +2.8% change among the D-LE schools (F-statistic = 7.3, p = 0.025). We found the same pattern in subset analyses that only included schools with the same dean at both time points (3 D-LE and 3 D-HE schools).

**Table 4:** Comparison of enforcement trajectory groups based on deans' and campus security chiefs' reports of enforcement levels over time.

	Deans' Enforcement Trajectory Groups				
	**Lower** **(n = 6)** **Mean (SD)**	**Higher** **(n = 5)** **Mean (SD)**		**F**	**p-value**

Deans' 1999 score	75.3 (4.0)	89.5 (7.8)		15.2	0.004
Deans' 2001 score	67.1 (5.8)	85.5 (10.8)		13.3	0.005
Deans' average score across years	71.2 (2.7)	87.5 (3.8)		70.9	<0.001
Change in past-30-days drinking rate	-4.4 (7.0)	-10.2 (11.8)		1.0	0.342
Change in past-2-weeks heavy episodic drinking rate	2.8 (10.1)	-18.5 (16.0)		7.3	0.025
Change in usual heavy episodic drinking rate *among past-30-days drinkers*	-13.7 (13.1)	-19.9 (10.1)		0.7	0.416
Change in past-2-weeks heavy episodic drinking rate *among those reporting no heavy drinking in high school*	-1.8 (38.1)	-22.7 (26.4)		1.1	0.318

	**Campus Security Chiefs' Enforcement Trajectory Groups**				

	**Lower** **(n = 5)** **Mean (SD)**	**Higher** **(n = 2)** **Mean (SD)**	**Increasing** **(n = 2)** **Mean (SD)**	**F**	**p-value**

Security chiefs' 1999 score	77.5 (5.7)	94.6 (0.9)	67.7 (8.7)	11.0	0.010
Security chiefs' 2001 score	74.0 (5.9)	90.0 (5.5)	86.3 (6.0)	6.7	0.030
Security chiefs' average score across years	75.8 (5.5)	92.3 (2.3)	77.0 (7.3)	6.8	0.029
Change in past-30-days drinking rate	-10.0 (12.8)	-3.7 (5.1)	-0.4 (6.4)	0.6	0.565
Change in past-2-weeks heavy episodic drinking rate	-6.5 (22.3)	-2.5 (22.4)	-3.9 (5.1)	0.0	0.969
Change in usual heavy episodic drinking rate *among past-30-days drinkers*	-16.9 (14.7)	-16.1 (18.3)	-17.8 (12.7)	0.1	0.994
Change in past-2-weeks heavy episodic drinking rate *among those reporting no heavy drinking in high school*	-18.0 (36.6)	10.3 (22.0)	14.2 (6.7)	0.9	0.439

We identified 3 distinct cluster groups based on security chiefs' scores over time. As with the deans' cluster groups, there was a "higher enforcement" group (C-HE) (n = 5) with mean scores >=80 across time, and a "lower enforcement" group (C-LE) (n = 2) with mean scores <80. A third group not found for the deans was the "increasing enforcement group" (C-IE) (n = 2), with initial chiefs' enforcement scores <80 but increasing to a mean >80 at the second time point. As shown in Table 4, we found little association between these trajectory groups and changes in student drinking rates.

## Discussion

Our study found that current (i.e., past 30 days) drinking rates and heavy drinking behavior among drinkers declined significantly during a period of increased restrictions on alcohol at Massachusetts public colleges and universities. While the great majority of students continued to report some drinking, study results suggest that *heavy *drinking may have declined, particularly among underage students and on-campus residents, for whom the school's alcohol policy may have greater impact. This apparent decline occurred despite an overall *higher *percentage in 2001 of students in our sample reporting heavy drinking during their last year in high school, a known risk factor for heavy drinking in college [16-19]. During the same time period, the national CAS found no significant change in the heavy drinking rate for public colleges and universities nationally (N = 61 schools excluding MA, heavy drinking rate 1999 48.3% vs. 2001 47.7%, p = NS (unpublished data)). However, there was a trend towards a decline (1999 52.8% vs. 2001 48.2%, p = .02) for students attending public colleges in the Northeast (Nelson T.F., personal communication). Therefore, the decline in MA may in part reflect, and have contributed to, this regional trend and our study findings may be confounded by other contextual factors that may have influenced student drinking patterns region-wide.

We were able to examine data from one school included in both our sample and the CAS sample prior to (1993, 1997) and after (2005) our study period, allowing us to look at trends for that school both leading up to and extending beyond our study period. This school's heavy drinking rates (weighted) steadily increased through 1999 (1993 58%, 1997 63%, 1999 71%), declined in 2001 (55%), and increased again by 2005 (67%) (unpublished data). The brief interruption of the upward trend immediately following the MA Board of Higher Education policy change could be, in part, due to the stricter alcohol policy, as well as to the increased attention to underage drinking generated by the two student deaths. However, the decline between 1999 and 2001 was short-lived at this school, and possibly statewide. Over time, memories can fade and enforcement may wane, leading to drinking behaviors returning to previous or even higher than previous levels.

Heavy drinking appeared to be less common over time as a usual drinking pattern among female students who drink, while there was no such change for men. Previous studies have found gender differences in both the likelihood of engaging in heavy episodic drinking, as well as the factors that increase the risk of heavy drinking in college [16,20-23]. Men tend to engage in heavy drinking more frequently than women, and to drink heavily more often than originally intended [24]. High-risk drinking among men may be more normative and entrenched, and therefore more difficult to reduce, requiring intervention strategies different from those that are effective for women.

College is a time of significantly increased drinking prevalence compared to the rates found among college-bound 12^th ^graders and non-college-attending young adults [1], indicating that many students take up heavy drinking when they arrive at college. We found declining trends between 1999 and 2001 in the rate of heavy drinking *uptake *among on-campus and underage students, groups that are more likely to feel the impact of stricter school alcohol policy enforcement and to be affected by sanctions.

We found no evidence of a rise in marijuana use as a possible unintended consequence of stricter alcohol policy enforcement. Trend data from the CAS and Monitoring the Future studies which showed a rise in marijuana use among college students between 1993 and 1999, a time of increased restrictions on alcohol use, caused concern about students substituting marijuana and other drugs for alcohol [22,25,26]. However, a report by Williams et al. found that government and campus anti-drinking policies (e.g., banning alcohol on campus, "happy hour" restrictions) were associated with lower rates of *both *alcohol and marijuana use, suggesting that demand for alcohol and marijuana may be complementary rather than substitutive [27]. While marijuana use rates changed little during our 2-year study period, we are not able to say whether use of other illicit drugs changed since analysis of other drug use trends was beyond the purview of this study.

Another possible unintended consequence of a stricter college alcohol policy is that students may go off campus to drink. We found a marginally significant increase in the prevalence of past-30-days DWI/RWID behavior among on-campus but not off-campus residents. This finding may indicate that students were more likely to go off campus to drink in response to the greater restrictions. However, we did not measure community characteristics and changes in local policies which could have contributed to this trend [28], and therefore cannot determine causality

In examining whether stricter policy enforcement was associated with changes in school drinking rates, we found that deans' perceptions of enforcement strength in 1999 were associated with greater *subsequent *declines in heavy drinking rates during the study follow-up period. In addition, schools with *consistently *high dean's enforcement scores appeared to have greater declines in past-2-weeks HED compared to schools with lower scores over time. In our previous cross-sectional study of 1999 data, however, we found that deans' enforcement scores had weak to moderate *positive *correlations with *concurrent *student drinking rates. In contrast, security chiefs' reports were strongly, and *negatively*, correlated with *concurrent *drinking rates [6] but showed lower associations with drinking rate *change *over time (i.e., higher enforcement scores were more associated with increases in drinking rates).

We hypothesize that the difference in our findings between the two studies may be due to the different perspectives that deans and campus security chiefs have on policy enforcement. Security chiefs are likely more knowledgeable about day-to-day enforcement activities and events, particularly those happening on evenings and weekends when students are more likely to drink. Their stricter enforcement may have a more immediate deterrent effect. On the other hand, deans may be more likely to base their enforcement perceptions on the number of students caught for violations, because they are usually charged with meting out disciplinary sanctions. In schools with more widespread drinking, deans may have more students to discipline, therefore resulting in a positive correlation between enforcement and drinking rates in a cross-sectional study. On the other hand, a more aggressive enforcement stance by deans may over time bring about changes in student perceptions and behavior, with fewer students *initiating *heavy drinking in college or engaging in heavy drinking as a usual behavior.

Our previous study [6] found that students' perceptions of enforcement were positively correlated with their own drinking behavior (i.e., students who drank were more likely to report strong enforcement compared to non-drinkers). In the follow-up study, students' perceptions of enforcement had little or no association with changes in drinking rates over time. Given the biased perspective that may result from students' susceptibility to getting sanctioned for drinking, students may not be the best informants about alcohol policy enforcement.

This longitudinal study represents an important extension of our previous cross-sectional study, allowing us to investigate the prospective relationship between alcohol policy enforcement levels and changes in rates of student drinking and associated behaviors across a statewide system of schools with a uniform alcohol policy. Our study adds to the growing body of work that suggests that environmental strategies addressing underage alcohol use can help to lower high-risk college drinking and associated behaviors [29-33]. In particular, multi-strategy efforts, such as that promulgated by the new MBHE policy, which combine multiple environmental strategies with individual student-focused interventions, were associated with reduced underage drinking, driving after drinking, and secondhand effects of student drinking [29,31,34]. For example, in their evaluation of the Matter of Degree program, a multi-strategy environmental initiative to reduce college drinking and related harms, Weitzmann and colleagues found that communities implementing a high number of interventions and programs to address alcohol availability, legal sanctioning, parent and peer influences, etc. significantly reduced their college student heavy drinking rates, while those with low implementation did not [34]. In addition, Toomey and colleagues cautiously suggest, based on their recent review of the extant research on the effects of environmental policies on college drinking, that a multi-pronged strategy involving campus-community collaboration, more alcohol-control policies, and stronger policy implementation and enforcement, may be effective; however, they point out that most studies had methodological issues such as a cross-sectional observational design and lack of multiple sites for comparison and further work is needed to more rigorously assess cause and effect [29].

This study had several strengths such as a longitudinal follow-up; inclusion of the assessment of multiple perspectives of alcohol policy enforcement; the use of multi-item enforcement measures that examined a range of enforcement activities, experiences, and venues; and the evaluation of alcohol use and related outcomes, as well as potential unintended consequences of a stricter alcohol policy (i.e., increased driving while intoxicated/riding with an intoxicated driver, and marijuana use). Our study also had a number of limitations. The number of schools was small, limiting the power of school-level analyses; we examined only two points in time; and we relied on self-reported rather than observational measures. In addition, the student survey response rates were generally low (range 38%-54%), raising concerns about potential selection bias and inaccurate estimates of student drinking rates. In examining whether heavy drinking prevalence estimates were associated with response rates, we found low correlations in both baseline and follow-up years. We also used direct standardization of the two years' samples to control for demographic differences in our comparisons of the two time points. Of greater concern is that student self-selection into particular schools *because *of the drinking policy may confound the association between policy enforcement and student drinking rates. If a stricter alcohol policy in the MA public colleges caused more drinking students to choose alternatives (e.g., private schools) over the public colleges, thus resulting in lower drinking rates in 2001, we would expect to find lower rates of high school HED among students in 2001 compared to 1999. Instead, we found the opposite, with *more *students in the 2001 sample reporting HED in high school than in 1999. Finally, we did not assess other influential environmental or individual factors that may confound the relationship between enforcement measures and student drinking trends (e.g. social marketing campaigns, student education and counseling, provision of alcohol-free alternative activities, community and state policies and regulations, alcohol pricing and promotion, and ease of access) [21,29,34-36]. We are also unable to identify the factors underlying the differences in administrator perceptions of policy enforcement such as different response biases that deans and campus security chiefs may have had. Because of these limitations, we advise caution in inferring a cause-effect relationship from our study results.

Future studies examining college alcohol policy enforcement should include many more schools, survey other key informants such as residence hall assistants and resident directors, include direct observation of campus enforcement practices (e.g., how often student ID's are checked at campus events, how frequently student bags are searched when entering dorms, etc.), and chronicle other on-campus and community-wide efforts to reduce student drinking.

## Conclusions

Our study findings suggest that an aggressive enforcement stance by deans, and other such college leaders, may be an important element of an effective college alcohol policy and be associated with reductions in student high-risk drinking rates over time, perhaps through reduced *uptake *of heavy drinking in college. A unified stance among college administrators of aggressive policy enforcement and action around drinking violations, and greater awareness of and involvement in enforcement by college leaders, e.g., through giving reminders at campus events and residence meetings, may help to set a tone on campus which discourages underage and heavy drinking by students. Our study findings also suggest the need for a *consistently *strong enforcement stance in order for effects to appear over time. While enforcement of alcohol policies may be challenging, colleges' multi-level efforts to address student drinking, when properly implemented and consistently enforced by college staff working in unison at all levels could eventually help to lower rates of students' heavy drinking, and therefore lower the morbidity and mortality among our nation's most important resource - its young people.

## List of abbreviations used

The following abbreviations were used: CAS: Harvard School of Public Health College Alcohol Study; MBHE: Massachusetts Board of Higher Education; T1: Time 1 data collection; T2: Time 2 data collection; HED: Past-2-weeks heavy episodic drinking defined as >=5 drinks in one occasion for males, >=4 for females; UHED: Usual heavy episodic drinking during the past 30 days; DWI/RWID: Driving while intoxicated/Riding with an intoxicated driver; B-H procedure: Benjamini-Hochberg procedure for controlling the false discovery rate; D-HE: Deans "higher enforcement" group; D-LE: Deans "lower enforcement group; C-HE: Campus security chiefs "higher enforcement" group; C-LE: Campus security chiefs "lower enforcement" group; C-IE: Campus security chiefs "increasing enforcement" group.

## Competing interests

The authors declare that they have no competing interests.

## Authors' contributions

SKH, LS, SVH, HW, and JRK participated in the conceptualization and design of the study, development of the measurement tools, and planning and review of the statistical analyses. SKH, LS, SVH, and JRK participated in the oversight of the implementation of the study, and SKH and LS performed the statistical analyses. SKH drafted the manuscript and all authors read and approved the final manuscript.
